# Regulatory Role of Circadian Clocks on ABA Production and Signaling, Stomatal Responses, and Water-Use Efficiency under Water-Deficit Conditions

**DOI:** 10.3390/cells11071154

**Published:** 2022-03-29

**Authors:** Yousef Yari Kamrani, Aida Shomali, Sasan Aliniaeifard, Oksana Lastochkina, Moein Moosavi-Nezhad, Nima Hajinajaf, Urszula Talar

**Affiliations:** 1 Experimental Biophysics, Institute for Biology, Humboldt-University of Berlin, Invaliden Str. 42, 10115 Berlin, Germany; 2 Photosynthesis Laboratory, Department of Horticulture, Aburaihan Campus, University of Tehran, Pakdasht P.O. Box 33916-53755, Tehran, Iran; aida.shomali@ut.ac.ir (A.S.); moein.moosavi@ut.ac.ir (M.M.-N.); 3 Institute of Biochemistry and Genetics, Subdivision of the Ufa Federal Research Center of the Russian Academy of Sciences, Pr. Oktyabrya, 71, 450054 Ufa, Russia; oksanaibg@gmail.com; 4 Chemical Engineering Program, School for Engineering of Matter, Transport, and Energy, Arizona State University, Tempe, AZ 85287, USA; nhajinaj@asu.edu; 5 Institute of Plant Genetics, Polish Academy of Sciences, Strzeszyńska 34, 60-479 Poznań, Poland; utal@igr.poznan.pl

**Keywords:** ABA signaling, abiotic stress, central oscillator, circadian clock, plant fitness

## Abstract

Plants deploy molecular, physiological, and anatomical adaptations to cope with long-term water-deficit exposure, and some of these processes are controlled by circadian clocks. Circadian clocks are endogenous timekeepers that autonomously modulate biological systems over the course of the day–night cycle. Plants’ responses to water deficiency vary with the time of the day. Opening and closing of stomata, which control water loss from plants, have diurnal responses based on the humidity level in the rhizosphere and the air surrounding the leaves. Abscisic acid (ABA), the main phytohormone modulating the stomatal response to water availability, is regulated by circadian clocks. The molecular mechanism of the plant’s circadian clock for regulating stress responses is composed not only of transcriptional but also posttranscriptional regulatory networks. Despite the importance of regulatory impact of circadian clock systems on ABA production and signaling, which is reflected in stomatal responses and as a consequence influences the drought tolerance response of the plants, the interrelationship between circadian clock, ABA homeostasis, and signaling and water-deficit responses has to date not been clearly described. In this review, we hypothesized that the circadian clock through ABA directs plants to modulate their responses and feedback mechanisms to ensure survival and to enhance their fitness under drought conditions. Different regulatory pathways and challenges in circadian-based rhythms and the possible adaptive advantage through them are also discussed.

## 1. Introduction

Seasonal changes are the result of the tilt in the Earth’s axis, which causes differences in the duration and intensity of sunlight. Solar irradiance and its spectral composition are modified by diurnal, seasonal, and latitudinal changes in spectral irradiance reaching the Earth’s surface. These changes depend on solar elevation angle, composition of the traversed air mass, and, partially, on spectral reflectance from the ground surface. Fluctuation in the lighting environment, canopy architecture, and other environmental factors determines the incident light intensity on the leaves, which in turn strongly impact many physiological processes including photosynthesis [1].

Plants undergo diurnal oscillations that are generated and maintained by an endogenous system known as the circadian clock [2]. The circadian system is a complex, inter-connected, and reciprocally regulated network. The core oscillator consists of many coupled feedback loops in plants. Circadian rhythms are governed by a molecular clock system that synchronizes plant function with daily light and temperature cycles to maintain homeostasis [3], and the efficient functioning of plants depends on the proper operation of the circadian clock system. In fact, many plant processes follow a rhythmic sinusoidal pattern over the course of a 24 h period, in perfect sync with the diurnal cycle. These oscillations persist in the absence of environmental stimuli (under continuous light and constant temperature), and can be sustained for several weeks [4,5].

Synchronization of physiological processes with external light–dark cycles has many advantages for plant survival, growth, and compliance with environmental constraints [2,3]. In *Arabidopsis*, plant fitness and survival are improved when the free-running period of the circadian clock matches the total duration of the light–dark cycle [6] and plants having matched clock periods with the environment photosynthesize more, and survive better, than those with circadian periods that are uncoordinated with environmental cues [2,3]. Interestingly, physiological processes such as photosynthesis have been also argued to largely affect the entrainment of the circadian clock in *Arabidopsis*. Induction of carbohydrate synthesis during daytime is the result of activation of light-dependent reaction of photosynthesis. Their depletion at night is the result of their utilization to prevent starvation before dawn due to respiration [7,8]. These are indicative of the beneficial impact of circadian clocks on plant growth and survival and their interconnection with other physiological processes. However, some doubts exist about the indispensable role of circadian clocks in survival and fitness since arrhythmic mutants are also able to survive [9]. Moreover, most researchers investigated the role of the circadian clock in stress response from a molecular aspect and the physiological mechanism underlying the role of the clock in adaption to stress conditions is less discussed.

The circadian system is a complex, inter-connected, and reciprocally regulated network. The core oscillator consists of many coupled feedback loops in plants. A circadian oscillator has been proposed to function as the key modulator of plant responses to environmental cues. Circadian clocks direct plant defense mechanisms during gradual exposure to stresses to fit better to the changes in environmental conditions [10].

Drought stress occurs when the plant’s water loss exceeds its roots’ ability to absorb water and the plant’s water content is low enough to interfere with normal plant operations [11]. This is a major constraint that restricts the development and efficiency of crop production. Tremendous efforts have been devoted to develop crops having boosted resilience to drought. To this end, considerable attention has been paid to abscisic acid (ABA), which plays a pivotal role in drought tolerance by tuning the stomatal movements and stress-responsive gene expression [11,12,13,14]. The molecular mechanisms of the plant circadian clock for assisting plant fitness and regulation of drought-stress responses through ABA are composed of not only transcriptional but also post-transcriptional regulatory networks.

This review was aimed at discussing the mechanisms through which circadian clocks play a role in shaping a response to drought stress. We will focus on the involvement of circadian clock components in ABA-dependent response to drought stress. The possible adaptive advantages through the clocks will be discussed regarding the interrelationships between circadian clocks, ABA production and signaling, stomatal responses, and water use.

## 2. How Does the Circadian Clock Work?

The clock helps plants adapt to the diurnal cycle by anticipating daily alterations in the environment and consequently the coordination of cellular processes [15]. However, the clock’s specification to tissues and cell types has been neglected in the majority of studies. Nevertheless, the clock’s main and intermediate components are portrayed through recent studies using forward and reverse genetic tools [16,17,18,19].

Different transcriptional networks interact with intrinsic clock components and shape a more complicated molecular network controlling the core of the circadian clock. Furthermore, complementary layers of regulation including posttranscriptional gene regulations enhance the robustness of the time-keeper’s oscillations [20,21]. The central oscillator is synchronized with, or entrained to, environmental time clues such as diurnal changes in light and temperature conditions [22]. Components of this central oscillator regulate gene transcription downstream of the clock in order to drive the rhythmicity of physiological processes [23].

**Figure 1:**
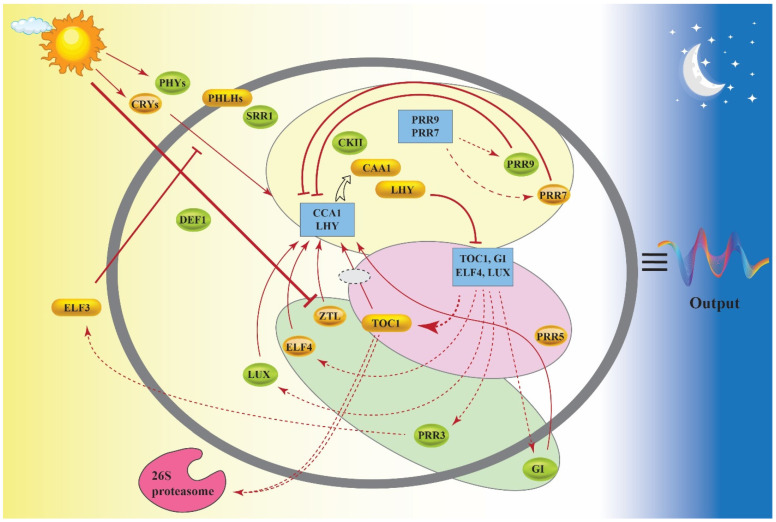
Feedback loops within the internal oscillators. Pink loop: the transcriptional feedback loop contains the morning-phased transcription factors CCA1 and LHY, and negatively regulates TOC1. Yellow loop: consists of PRR7, PRR9, CCA1, and LHY. Green loop: represents ZTL, which is the negative regulator of TOC1, also regulated by the establishment of GI and PRR3. (Rectangles: genes; Ovals: proteins; Dashed lines: transcription/translation; Solid lines: protein activity; Lines ending in arrows: stimulatory function; Dashes: negative action of the corresponding proteins).

Analysis of genome-wide gene expression revealed that the core components of the clock are conserved between plant species [24,25]. In *Arabidopsis*, the circadian clock is composed of a negative feedback loop comprising three inhibitory steps [26,27]. The cycle begins with the expression of two single MYB-domain transcription factors known as CIRCADIAN CLOCK ASSOCIATED 1 (CCA1) and LATE ELONGATED HYPOCOTYL (LHY), which act to repress the expression of all other clock-associated genes. The LHY and CCA1 proteins also act to downregulate their own expression, allowing the expression of a set of Pseudo-Response Regulator genes (*PRR9*,*7*,*5*, and *PRR1*, also known as *TOC1*) to rise over the course of the day. The PRR proteins act to further repress the expression of LHY and CCA1 during the day and early night. This repression is lifted late at night through the action of ELF3, ELF4, and LUX. These proteins form an evening complex which shuts down the expression of the *PRR* genes, allowing expression of *LHY* and *CCA1* to resume at dawn and the cycle to restart (Figure 1).

TOC1 activity is further modulated at the post-translational level through the action of the F-box protein ZEITLUPE (ZTL). The ZTL protein senses blue light through its LOV domain, and forms a complex with GIGANTEA. However, in darkness, ZTL is released and interacts with TOC1, targeting it for ubiquitination and degradation by the 26S proteasome [28].

## 3. Circadian Clock Plays a Central Role in Plant Fitness

The endogenous clock conferring plants a fitness advantage enables them to anticipate periodic environmental changes and adapt correspondingly, thus contributing to the physiological and developmental homeostasis of plants [29]. This happens initially through communicating an estimate of the time of day to circadian-regulated features of the cell and later to transcriptional regulation [30,31]. Previous reports contradicted this hypothesis as they concluded that the endogenous clock may indirectly be involved in adaption as part of correlation with other adaptive traits and are unemployed for adaption in new generations compared to their ancestors [32]. Moreover, despite several documents supporting the regulatory role of the circadian clock in plants’ fitness, there are some reports challenging the evolutionary role of molecular regulation based on the circadian clock. For instance, comparative transcriptome analysis showed that clock-based regulation of transcriptomes is manifested more in unicellular organisms than in modern plants [33]. Regarding this, recent molecular studies revealed that, during the evolution, clock-regulation of transcriptome is reduced from 90% of the transcriptome in unicellular organisms such as algae [34] to 50% of the transcriptome in evolved and flexible species such as modern plants [35,36]. However, studying a variant population of *Arabidopsis* presenting a range of circadian rhythm periods, it was revealed that plants whose internal clock more closely matched the environmental T-cycle (light⁄dark cycle lengths) were properly adapted and produced more viable offspring in subsequent generations [29]. This discrepancy invites a more in-depth investigation of the underlying mechanisms of direct or indirect involvement of the circadian clock in plants’ fitness. Therefore, in the following section, we will discuss the involvement of the circadian clock in plants’ resilience to drought stress via triggering photoprotection, stomatal movements, drought stress, and water-use efficiency (WUE) through the involvement of ABA.

## 4. Circadian Clock Enhances Fitness by Controlling Photoprotection via the Xanthophyll Cycle

Although light exposure is vital for plant growth and development, excess photoabsorption damages the photosynthetic apparatus and frequently imposes an inhibitory effect on photosynthesis, a process known as photoinhibition [37]. Plant photosynthetic machinery under excessive light generates toxic by-products of oxygen (O_2_), ROS, which leads to oxidative damage to the photosynthetic apparatus of the plants [38,39]. To cope with light stress, plants have developed several photoprotective mechanisms to avoid the absorption of excess light energy, thereby reducing photodamage. Plants are able to acclimate to different environmental conditions to alleviate the detrimental effects of excess light on growth and viability [40]. Under excess light conditions, plants may acquire a balanced state of photomorphogenesis (chloroplast avoidance movements and leaf anatomical modifications) to avoid the absorption of excess light energy and reduce photodamage through a feedback mechanism. Furthermore, it has already been demonstrated that xanthophyll cycles protect plants against oxidative stress generated by exposure to high light intensity. It is demonstrated that the xanthophyll cycle shields against oxidative stress generated by high light irradiation. At the molecular level, some carotenoids take part in quenching chlorophyll triplets and prevent the formation of ROS [41]. More precisely, a low pH in the luminal side of the thylakoid membrane activates the violaxanthin de-epoxidase (VDE) that converts violaxanthin (V) into zeaxanthin (Z), which facilitates light-regulated switching of PSII from a light-harvesting state to an energy-dissipating state under excessive light. Therefore, these cycles assist photo-acclimation of plants under varying light conditions [42].

Circadian regulation of physiological traits has been documented in a large number of studies concerning both species and photosynthetic syndromes [43,44]. However, clock responses to high light stress in plants are not yet clearly understood. Yarkhunova et al. (2018) showed that the disruption of clock function via mutations leads to shifts in Fv′/Fm′ (the chlorophyll fluorescence parameter reflecting the efficiency of photosystem II (PSII) photochemistry) and non-photochemical quenching (NPQ), such that wild-type plants have both higher Fv′/Fm′ and lower total NPQ, representing more efficient photosynthetic functionality [45]. NPQ consists of responses that occur over longer periods, allowing for acclimation to high light exposure, as well as short-term responses to rapid fluctuations in light. There are three types of NPQ categorized based on the time scales of their induction and relaxation: energy-dependent feedback de-excitation quenching (qE) [38], state transition-dependent quenching (qT), and photoinhibitory quenching (qI).

A high NPQ value indicates the absorption of excessive photons and shows the efficiency of the dissipation of excessive excitation energy into harmless heat. Transcriptomic studies have revealed that some genes coding the enzymes required for state transitions (e.g., STN7 protein kinase, AT1G68830, AT5G01920, and AT4G27800) are circadian-regulated [45,46], suggesting that the clock plays an important role in the synchronization of state transitions. It is worth noting here that quenching excess energy in a non-photochemical way, either by state transition (qT) or photoinhibition (qI), shows correlations with the circadian period, and neither the genes responsible for qE sites such as LHCII, CP29, and CP26 (e.g., AT1G19150, AT3G53460, AT4G10340) nor the genes associated with photoinhibition (e.g., AT1G77510, AT2G30950, AT3G19570) are under circadian control.

Galvez-Valdivieso et al. (2009) showed that abscisic acid (ABA) signaling is associated with extracellular ROS production and the expression of high light-responsive genes including RD20, HSP17.6B-C1, and HSP17.6C-C1 [47]. ABA content of *Arabidopsis* leaves increases following 15 min exposure to high light and low humidity in either attached and desiccated leaves, however, no significant differences between NPQ of the nced3 mutant with low ABA content and wild-type cells have been reported [48].

**Figure 2:**
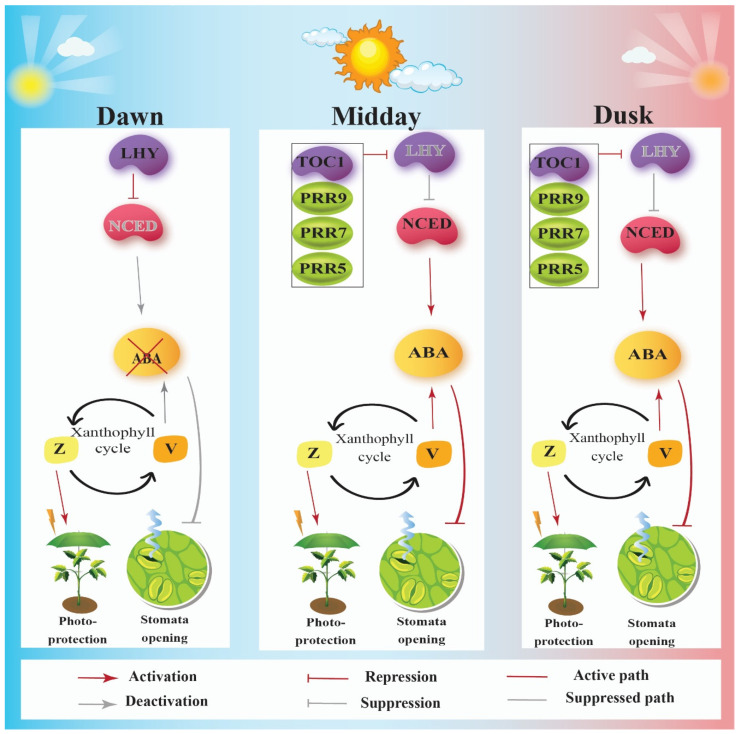
Regulatory role of circadian clocks on the biosynthesis of abscisic acid (ABA) and regulation of photo-protection, or on the PIF4 expression for regulation of thermo-morphogenesis, during different times of the day.

In contrast, using a new transcriptomics approach, Huang et al. (2019) highlighted three important findings: first, the ABA biosynthetic genes, including 9-cis-epoxycarotenoid dioxygenase (NCED) 3, NCED5, and NCED9, are upregulated after 0.5 h of high light exposure. Second, the ABA levels increased slightly after 0.5 h of high light, increased sharply after 6 h of high light exposure, then reached a plateau for the subsequent exposure. Third, the most important finding with respect to our discussion was indicative of severe hypersensitivity to 24 h high light exposure in ABA biosynthesis-defective double mutant *nced3 nced5* [49]. The mutant plants had bleached cotyledons with larger bleached areas compared to their wild-type counterparts. In brief, these results not only suggested that ABA might be a signal for triggering response to high light [47,50] in the short term, but it may also be a signaling component in the continuous high level of ABA content during different durations of high light exposure (Figure 2). The high light-hypersensitive phenotype of *nced3 nced5* double mutants is indicative of the important role of ABA in middle- and long-term high light-stress responses. However, the mechanism linking ABA accumulation to photosensitivity remains to be investigated in the future. One possible scenario could be the dissipation of light energy due to either a defect in Z or PSBS accumulation, which leads to photosensitivity; therefore, further investigation by quantifying Z content or PSBS accumulation under high light in nced3 nced5 mutant could shed light on the photoprotection mechanism.

Another interesting study in unicellular green algae *Chlamydomonas reinhardtii* showed that the *LUX* homologue ROC75, which functions as a key component of the central circadian clock, acts to attenuate expression of the major qE effector, LIGHT-HARVESTING COMPLEX STRESS-RELATED PROTEIN 3 (LHCSR3), under blue and red light [51,52]. These findings on the photoprotective role of ROC75 may bring a new viewpoint for plant researchers to investigate the role of the circadian clock in photoprotection.

## 5. Circadian Clock Enhances Fitness by Assisting Adaptive Stomatal Movements

Stomata are the gas exchange gates of plants. Considering the diverse roles of stomata in plant responses to environmental cues, they enable plants to cope with changing environments despite the sessile nature of plants [53]. The guard cells have the ability to integrate a wide variety of signals to regulate stomatal movements in order to establish a balance between CO_2_ uptake and water loss [54]. The clock endogenous oscillator regulates the sensitivity of the guard cells in response to exogenous stimuli that are reflected by stomatal movements [55]. The response of stomata to exogenous cues depends on the phase of the circadian clock at the time of stimulation. In other words, circadian-regulated processes discriminate between signaling pathways according to the time of day, which guarantees the phase-appropriate response of the guard cells to exogenous stimuli. Thereby, the clock provides a mechanism for signal processing and transduction in response to stimuli, which is known as the ‘gating’ mechanism [55]. Several experiments revealed that the gating mechanism is not only affected by the influence of the clock on the sensitivity of output pathways, but also determines the sensitivity of the feedback pathways back into the clock’s internal oscillator. For instance, the clock regulates ABA biosynthesis, and gates downstream responses [56]; in turn, ABA induces TOC1 expression 5–10 h after dawn, suggesting a role for TOC1 as both the regulator of the internal oscillator’s pace and the modulator of downstream process kinetics [57,58].

An increasing number of reports are evidencing the circadian gating of responses to various stimuli, including ABA [59], auxins [60], light [3], and pathogens [61]. The clock was also shown to play specific roles in the regulation of ABA signaling at numerous points of the network, including ABA biosynthesis, activation, transport, and metabolism [56,62,63].

This adaptive role of circadian clocks to improve growth and survival can be related to the regulation of carbon metabolism [64]. Here, we focus on the role of the circadian clock and light/dark cycles on the ABA metabolism as the main phytohormone involved in the regulation of stomatal aperture influencing carbon metabolism through photosynthesis. Although it is indicated that vapor pressure deficit (VPD) is the driving force to induce stomata to open/close, light is considered as a signal that promotes stomatal opening, and conversely, darkness causes its closure [65].

**Figure 3:**
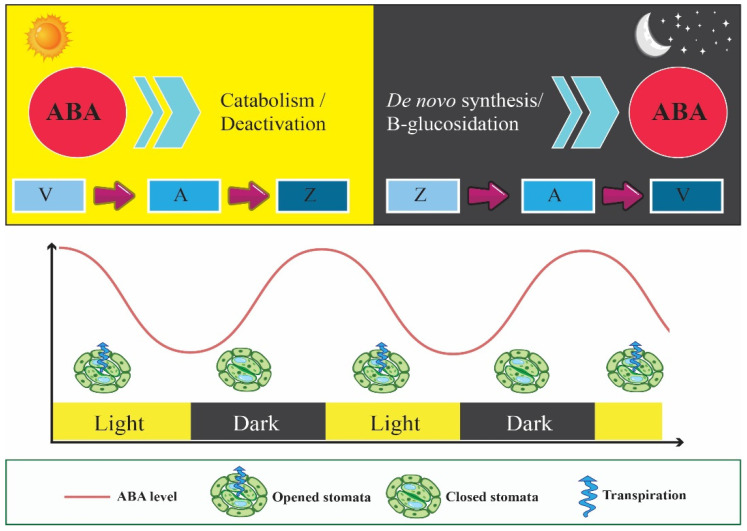
Circadian rhythms in ABA levels and stomatal opening/closure under non-stressful conditions. Light period promotes the conversion of violaxanthin (V) into antheraxanthin (A) and then zeaxanthin (Z) in the xanthophyll cycle, leading to ABA catabolism/deactivation and stomatal opening, while darkness favors the production of V as the ABA biosynthesis precursor, and stomatal closure.

During exposure to light and under normal conditions, ABA levels decrease as the result of three mechanisms: (i) excitation of chlorophyll molecules in the photosystems and activation of the photosynthesis-triggered conversion of violaxanthin (V) to antheraxanthin (A), and then zeaxanthin (Z), in the xanthophyll cycle. Since V and other cis-epoxycarotenoids are derived as the precursor for ABA biosynthesis, the pool of ABA precursors would be restricted during the day; (ii) glucose conjugates to ABA during the light period, which results in de-activation of ABA; and (iii) activation of ABA 8′-hydroxylases, which are activated by elevated O_2_ and reduced CO_2_ levels due to mesophyll photosynthesis [12,66]. ABA 8′-hydroxylases degrade ABA to phaseic acid [67]. Consequently, ABA reaches its minimum levels during the daytime. This regulating mechanism is based on favoring phototropin-mediated activation of guard cell H^+^-pump leading to stomatal opening, acceleration of the photosynthesis process, and the production of carbohydrates. In the dark phase, the ABA 8′-hydroxylase activity would be restricted as the result of low O_2_ and high CO_2_ concentrations due to the cessation of photosynthesis and decreased NADPH pool, while the xanthophyll cycle is directed towards the production of V, which promotes ABA biosynthesis. Consequently, ABA levels increased during the dark period, leading to the closure of stomata (Figure 3) [66].

## 6. Circadian Clock Enhances Fitness by Controlling WUE through Affecting Stomatal Movements

The circadian clock’s oscillator bears adaptive advantages by providing plants a cellular measure of the daytime to improve water-use efficiency (WUE) [31]. In plants, WUE is a complex trait that is derived through integrative molecular and physiological networks. The central component of this network is the stomatal movements. An increasing number of studies have demonstrated that the endogenous circadian oscillator modulates the circadian rhythm of stomatal movement [30,68,69] and modulates the responses of stomatal guard cells to environmental cues [70,71]. Calcium (Ca^+^) signaling is one of the primary regulators of the osmotic stress response [11,72]. The circadian regulation of stomatal movement underlies clock-regulated Ca^+^ signaling. Coincidence of cytosolic Ca^+^ pick accumulation with high stomatal responsiveness to ABA in the afternoon provides a clock-regulated framework for the stomatal movement to maximize WUE in plants [73].

The abundance of transcripts encoding CCA1, LHY, TOC1, and GI showed circadian oscillations in the guard cells [29]. Previous research demonstrated that the short-period mutant toc1-1 shortens circadian waves of stomatal conductance [74], while the long-period mutant ztl-1 prolongs the rhythms of stomatal conductance [74,75], and the constitutive overexpression of *CCA1* alters the daily regulations of stomatal movement under continuous light, which increases the stomatal conductance throughout the photoperiod [3]. Accordingly, the clock endogenous oscillator within guard cells makes an important contribution to the daily regulation of stomatal opening [23].

The fact that the circadian oscillator contributes to both stomatal movement [3] and biomass accumulation [76], as well as the finding that mutations or over-expression of clock components (e.g., CCA1, TOC1, ELF3, GI, GRP7, PRR9, TEJ, TIC, and ZTL) resulted in altered WUE, allows the postulation that the clock might take part in long-term WUE and consequent plant fitness [23]. The circadian clock, through the mediation of stomatal movement, may also affect the daily pattern of net exchange of CO_2_ [77]. Nocturnal opening of stomata may be an adaptive behavior providing high value of stomatal conductance for the time when it is accompanied with low temperature and low VPD, which maximize the potential of plants for photosynthesis. On the other hand, the diurnal closure of stomata reduces photosynthesis at the expense of preventing water loss in hot hours of the day [78]. The circadian dependence on stomatal aperture is also influenced by the response of stomatal aperture to ABA, VPD, and time of the day, showing a peak in the afternoon [59,79]. This clock-gated response to ABA was shown to be a conserved behavior that evolved through evolution [80].

Interestingly, the stomatal aperture is affected by several key genes that are involved in circadian-controlled pathways that mediate photoperiodic flowering [81,82] including FLOWERING LOCUS T (FT), GIGANTEA (GI), CO, and ELF3. FT induces stomatal opening in response to blue light. The upregulation of FT by GI and CO results in stomatal opening and its repression by ELF3 leads to stomatal closure [82,83]. The level of FT transcript shows a circadian rhythm parallel with an oscillating pattern of stomatal aperture under constant light conditions. Moreover, loss-of-function mutants of FT have shown no circadian rhythm under constant light and insensitive to blue-light activation of the H^+^-ATPase with reduced stomatal aperture [82]. Wide-open stomata and high H^+^-ATPase activity has been observed in all *elf3* loss-of-function alleles and also the *elf3 phot1 phot2* triple mutant under either darkness or blue-light exposure. Moreover, the level of FT mRNA was also increased in guard cells, which confirms that ELF3 is a negative regulator of stomatal aperture in response to light [84]. Accordingly, Chen et al. (2012) proposed that the associated role of ELF3 and FT with the circadian oscillator in the regulation of stomatal aperture may underlie signal conveyance from the circadian clock or its downstream and photoperiod-dependence of stomatal sensitivity to blue light [85].

AtMYB60 is a member of the *Arabidopsis* R2R3-*MYB* gene family, and acts as a guard cell-specific promotor of TOC1 and CCA1, which modulates the stomatal aperture [23]. The expression of AtMYB60 is induced in response to blue light and diurnal cues, while the level of its transcripts reduces in the dark [86]. Null mutants of atmyb60 are associated with constitutive reduction in stomatal opening and thus reduced wilting under water scarcity [85]. Another member of the *Arabidopsis* R2R3-*MYB* gene family, AtMYB61, also expresses in guard cells, however, it negatively modulates stomatal opening in response to light. The abundance of its transcript was shown to be maximized in the middle of the dark period coincident with reduced stomatal aperture, and considerably reduced during light periods coincident with a wide stomatal aperture [87]. In addition, the atmyb61 loss-of-function mutant was associated with a weak phenotype of wider-open stomata and also 0.5 °C-cooler leaf compared to the wild-type plants [87]. It has been indicated that stomata-specific expression of CsMYB61 in *Arabidopsis* resulted in an enhanced WUE [88]. Given these discrepancies about the functional role of MYB61 in stomatal movement and WUE, Simon et al. [23] investigated if the altered dehydration tolerance is related to the circadian oscillation of guard cells by analyzing constitutive overexpressed CCA1 and TOC1 plants using two guard cell-specific promoters (GC1 and MYB60). According to their report, MYB60::CCA1 and GC1::CCA1 transgenic plants had increased dehydration survival, whereas GC1::TOC1 and MYB60::TOC1 showed minimum levels of dehydration survival compared to the wild type, suggesting that guard cell-specific CCA-ox contributes to increase and TOC1-ox may decrease dehydration tolerance under constant light conditions [23].

## 7. Circadian Clock Enhances Fitness by Improving Plant Drought-Stress Responses

The circadian clock is tightly linked with abiotic-stress responses. It regulates the transcription of stress-involving genes, which facilitates adaptive responses. Additionally, metabolic and homeostasis balances are synchronized with the plant circadian clock (Sanchez et al., 2011). The interrelation between drought-stress signaling and the circadian rhythm has been depicted in different plant species [89,90]. The majority of drought-stress-responsive elements follow a rhythmic expression pattern which synchronizes the physiological response of plants with circadian regulation [90,91,92]. It was demonstrated that the transcription of a number of genes contributes to drought- and osmotic-stress responses in a diurnal pattern, including EARLY RESPONSE TO DEHYDRATION 10 (ERD10) and 7 (ERD7), COLD-REGULATED 15 B (COR15B) and A (COR15A), RESPONSE TO DESSICATION 29 A (RD29A), DEHYDRATION-RESPONSIVE ELEMENT-BINDING (DREB), and BASIC LEUCINE ZIPPER (bZIP) [90,93].

Genome-wide analysis of gene expression in *Populus simonii* under drought stress revealed that the abundance of LHY that is a central component of the clock is decreased under water-deficit stress [94]. Moreover, an interrelation between drought-responsive genes and the circadian clock has been proposed in soybean since several stress-responsive genes exhibited a clock-gated expression. In this plant, circadian rhythm is disturbed due to the downregulation of evening-specific components of the clock (TOC1, LUX, and ELF4 genes) [90]. Differential expression of clock genes under drought stress has been reported in soybean and the amplitude of PRR7 and TOC1 gene expressions was found to deviate from normal conditions. In addition, a drought-specific splicing pattern has been detected for PRR3, which may act cooperatively with PRR7 and TOC1 to provide energy homeostasis during drought-stress conditions [95].

During drought conditions, TOC1 binds to the promotor of ABA-related genes, ABAR/CHLH/GUN5, and regulates its clock-gated expression. Sequentially, the expression of *ABAR* is advanced through the induction of TOC1 by ABA. In addition, ABAR and TOC1 RNAi/overexpression lines were characterized with impairment in the induction of TOC1 by ABA, suggesting that reciprocal regulation between TOC1 and circadian expression of ABAR is crucial for ABA-dependent response to drought [57]. Besides, TOC1 is introduced as a molecular switch that links the clock with the plant drought-stress-response pathway [57]. Studying TOC1 as the main component of the clock in roots and shoots revealed that participation of the clock in drought fitness was tissue-specific, TOC1 expression in the root did not contribute to TOC1-dependent fitness responses, and TOC1-deficient lines failed to convert biomass to seed capsule [96]. Nevertheless, a TOC1-dependent manner was found in R/FR-related drought responses in Nicotiana attenuate shoots via transcriptomic analysis and screening of transgenic lines [96]. Mutation in GI, an evening component of the clock, showed hypersensitivity to drought due to defected stomatal closure and uncontrolled water loss in *Arabidopsis*, which is due to reduction in expression of NCED3 [97].

The obvious rhythmic expression trend with PEG treatment as well as qRT-PCR verification of PEG-induced expression of several members of the PRR gene family uncovered the involvement of GhPRR in drought-stress response in *Gossypium hirsutum* [25]. In *Arabidopsis*, expression of drought-stress-response genes including DREB1/CBF (DEHYDRATION-RESPONSIVE ELEMENT B1/C-REPEAT-BINDING FACTOR) was conferred in prr9 prr7 prr5 mutants [98]. Nakamichi et al. (2016) studied *Arabidopsis* transgenic lines constitutively expressing PRR5 fused to a construct of two tandem VP16 stringent transcriptional activation domains (PRR5-VP), which is a negative regulator of endogenous PRR function. Using genome-wide gene expression profiling, they revealed that in transgenic lines, genes related to water-deprivation responses and cold stress were up-regulated [70].

Plants respond to drought stress through complex networks including ABA-dependent and ABA-independent pathways [99]. A close overlap between the circadian clock-regulated transcripts and ABA has been reported [93]. This issue has attracted the attention of many scientists because ABA regulates many abiotic-stress responses, including drought, water stress, and frost [100]. Interestingly, microarray comparative transcriptome analysis revealed an extensive overlap between circadian datasets and ABA, suggesting that many of the key genes that are involved in ABA biosynthesis and signal transduction are under the control of the circadian clock [93,101].

The regulatory role of circadian clocks in drought-stress signaling was first revealed through the transcriptome analysis in Arabidopsis. This confirmed the ABA-induced TOC1 expression through a clock-gated process, which is indicative of the coincidence of ABA-dependent stress response pathway with circadian rhythm [57].

Emerging evidence has indicated the involvement of the main component of the clock-TOC1 in ABA-induced stomatal movement. Defects in ABA-dependent stomatal closure in TOC1 miss-expressing plants are another potential clue for the circadian-regulated nature of drought-induced stomatal closure [57]. It was also shown that in wild-type plants, ABA-induced TOC1 expression occurs during the day whereas, the same response is absent at night. ABA-induced TOC1 expression acts as a molecular switch that bears adaptive advantages through attuning the physiological response of plants under drought stress, in such a way that relative amounts of ABA make a balance between the mechanisms involved in preventing water loss and relieving the mechanisms at the expense of proceeding photosynthesis [57]. This results in preventing water loss during hot hours of the day under water-deficit conditions [102]. Therefore, to confirm the involvement of the circadian clock in response to drought stress, the reciprocal relation between the ABA-dependent pathway of stress response and the circadian clock is hypothesized.

ABA is regulated in a clock-dependent manner. The circadian oscillator components regulate the expression and post-translational modification of ABA synthesis and signaling [103]. Adams et al. (2018) revealed that the coupling between the circadian clock and ABA pathways most likely makes an important contribution to plant performance under drought- and osmotic-stress conditions. They showed that inhibition of NCED gene expression by LHY results in reduced accumulation of ABA in the morning. Their studies further revealed that LHY ensures accumulation of ABA at dusk under severe water deficit in leaves [104]. In fact, this mechanism can be considered as an anticipatory function, enabling plants to activate drought-tolerance processes at the time when needed. On the other hand, LHY also acts to enable responses to ABA in the dawn, ensuring high-amplitude responses to unexpected hot or dry conditions in the daytime. Therefore, considering the theoretical role of light/dark cycles as the provokers of opening/closure response of the stomata, environmental cues result in a high/low ABA level (higher/lower than a threshold level for a specific duration of time). This can disturb normal patterns of stomatal opening/closure as an example of the circadian rhythm response. However, the anticipatory function of ABA in these responses is needs to be elucidated in detail.

Environmental stress can also have an impact on the abundance of clock-associated proteins at the post-translational level. For example, salt stress induces the proteasomal degradation of the GI protein, hence decreasing the amplitude of its oscillations [7]. This enables plants to synchronize their physiological behavior and response (e.g., stomatal movement) to time of the day by clock-dependent regulation of transcription factors and post-transcriptional regulations.

## 8. Circadian Clock Interacts with ABA Signaling Pathway

**Figure 4:**
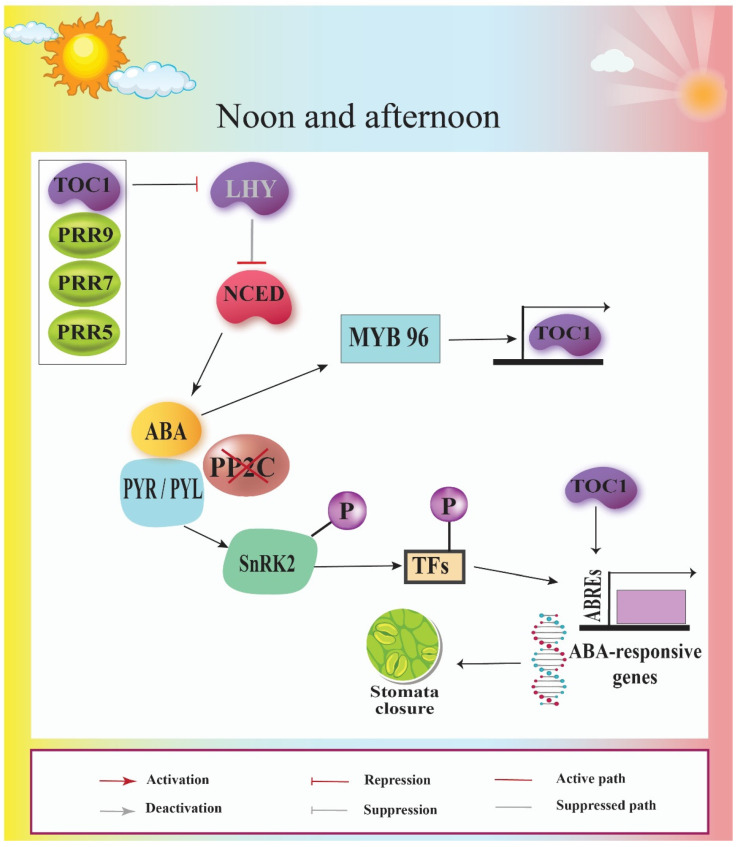
Scheme representing the signaling network of ABA regulatory pathway at the core of the circadian oscillator. In the morning, the biosynthesis of ABA is blocked through the negative impact of LHY on the NCED as the rate-limiting component of the ABA biosynthesis pathway. Later, following noon, when the plant gets rid of the negative impact of LHY on the NCED, ABA is present and its signal transduction pathway would activate the ABA-responsive genes, leading to stomatal closure.

According to molecular evidence, ABA signaling is under the control of the circadian clock. A comparison between the circadian clock and ABA-responsive transcriptional profile revealed 40% of ABA-responsive genes are controlled by the circadian clock [105]. Since the plant circadian clock has a role in drought- and osmotic-stress tolerance [25,30,73,106], underlying its mechanism of action in plant stress response is a valuable approach in the enhancement of plants’ performance under stress conditions [7]. It has been shown that tolerance to stressors would be disturbed in plants with impaired central oscillator activity [107,108,109]. Transcription of a number of genes contributing in the drought- and osmotic-stress responses, including ERD10 and 7 (ERD7), COR15B and A (COR15A), RD29A, DREB, and bZIP, follow a diurnal pattern [90,93]. As indicated before, the circadian clock also regulates the production of ABA, suggesting that the clock may function to intensify responses to heat, drought, and osmotic stresses during the day by regulating ABA production [110,111]. ABA production and concentration show strong daily changes which culminate in the afternoon. NCED3 is the most highly expressed NCED enzyme in root and stem tissues, is highly induced under drought conditions, and plays a major role in ABA production in response to water deficit [112,113]. Multiple ABA receptors have been found, but only one family of these proteins, known as pyrabactin resistance (PYR)-like (PYL) or regulatory component of ABA receptor, has had its downstream signal transduction pathways characterized (RCAR) [114,115]. When ABA binds to the PYL/RCAR receptor, the co-receptor protein phosphatase 2C (PP2C) is inactivated and a specific group of kinases called SNF1-related kinases 2 is activated SNRK2 [115,116]. SNRK2 kinase phosphorylates ABA-responsive transcription factors that bind to ABA-responsive elements (ABRE) within the promoter of ABA-responsive genes and regulates their expression [117]. Furthermore, the MYB transcription factor LHY modulates clock gating of ABA signaling by directly binding to the promoters of several genes involved in ABA production and signaling [56,97]. LHY inhibits the expression of ABI1 and ABI2 protein phosphatases [56] as well as repressing NCED3 expression, which suggests LHY may act as a negative regulator of ABA accumulation (Figure 4). ABl1 and ABl2 act as regulatory subunits of the ABA receptor (PYR / PYL) and suppress downstream responses in the absence of ABA. Furthermore, LHY controls the periodic buildup of ABA, ensuring that the phytohormone reaches its peak around dusk, when leaves are most dehydrated [104]. LHY also potentiates ABA responses in the morning, ensuring maximum responses to unanticipated hot or dry environments later in the day (Figure 4) [56]. On the other hand, at dawn, the stomatal opening exhibits just a small response to ABA, but ABA reaction peaks in the afternoon.

In the absence of PRRs such as toc1-2 (PRR1/TOC1, TIMING OF CAB EXPRESSION 1) and prr975 (PRR9, 7, and 5), reduction in leaf water loss would occur. TOC1 binds directly to the promoter of the ABA-related gene (ABAR/CHLH/GUN5) in the evening, repressing its expression [57]. MYB96, a drought- and ABA-inducible MYB transcription factor, is regulated by TOC1 and directly binds to the TOC1 promoter to positively control its expression [63] (Figure 4). Plant drought tolerance is determined by the MYB96–TOC1 reciprocal regulation. CCA1 binds to the promoter of MYB96, which influences its circadian expression. However, in order to understand how circadian clocks coordinate the physiological adaptations of growth and stress resistance, changes in plant biomass and essential agronomic variables must be investigated together. Table 1 shows the drought-response findings of various plant species and circadian clock genes.

An interrelation between drought-responsive genes and the circadian clock has been proposed in soybean since several stress-responsive genes show a clock-gated expression, and also circadian rhythm was disturbed due to down-regulation of evening-specific components of the clock (TOC1, LUX, and ELF4 genes) [90]. Furthermore, considering the expression of clock genes in rice under drought revealed that the transcription of OsCCA1/LHY increased at 4 a.m., and also the expression of OsPRR1/TOC1 was disrupted under drought [118]. Differential expression of clock genes under drought stress has been reported in soybean and the amplitude of PRR7 and TOC1 gene expressions was found to deviate from the normal condition. In addition, a drought-specific splicing pattern was detected for PRR3, which was suggested to be a modification for cooperative acting with PRR7 and TOC1 to provide energy homeostasis during drought-stress conditions [95].

B-Box (BBX) is one of the largest families of light-regulated proteins. The role of BBX in stress tolerance and light-mediated regulation of circadian signaling has been revealed [120,121,122]. CO/BBX1, BBX5, and BBX21/STH2 are known for their functions in ABA signaling [123,124,125]. Hypersensitivity during germination and cotyledon greening in response to ABA and osmotic stress are shown by *bbx5* mutants [123]. It has been reported that *CmBBX19* in *Chrysanthemum morifolium* modulates drought tolerance by inducing changes in expression of ABA-dependent pathway genes, *CmRAB18* and *CmRD29B*, and expression of *CmBBX19* is down-regulated by ABA [126]. Furthermore, apple *MdBBX10* gene is induced by exogenous ABA application in apple roots and leaves. Interestingly, MdBBX10 protein enhances drought-stress tolerance, when overexpressed in *Arabidopsis* [121]. It is worth mentioning that ABRE is found in the promoter region of many *BBX* genes among all plant species with identified and characterized *BBX* gene family [127,128,129]. In addition, BBX21/STH2 connects the light and ABA signaling by interacting with HY5 (ELONGATED HYPOCOTYL 5) and ABI5 (ABA INSENSITIVE 5) [125,130]. However, further investigation is necessary for the elucidation of the exact role of *BBX* genes in ABA signaling. Furthermore, there is evidence of the interplay between *BBX* and the circadian clock mechanism. Transcriptional analysis has shown that *AtBBX18*, *AtBBX19*, *AtBBX22*, *AtBBX24*, and *AtBBX25* genes in *Arabidopsis* are under regulation of the circadian clock [131,132]. Furthermore, in potato, several *StBBX* genes (*StBBX1*, *StBBX4*, *StBBX9*, *StBBX14*, *StBBX16*, *StBBX20/StBBX24*, *StBBX21*, and *StBBX25*) are shown to be under circadian regulation [122]. In fact, in the promoter regions of clock-controlled genes, specific *cis*-elements “CAANNNATC”, named circadian, associated with the circadian regulation, were found [122,133]. Bioinformatics analysis revealed that the circadian motif is localized in promotors of many potato *BBX* genes, including: *StBBX3*, *StBBX5*, *StBBX6*, *StBBX8*, *StBBX9*, *StBBX10*, *StBBX15*, *StBBX18*, *StBBX20/StBBX24*, *StBBX21*, *StBBX22*, *StBBX25*, *StBBX27*, *StBBX29*, and *StBBX30*, and those results are significantly divergent from results obtained from expression analysis. The reason may be that there is another, yet unknown, *cis*-regulatory element responsible for circadian regulation, or *StBBX* genes are regulated by some other molecular mechanism. Interestingly, the transcription factor *StZPR1*, belonging to the zinc finger family type C4, which has been identified recently, binds to the “CAACAGCATC” motif, defined by the term CIRC (circadian-regulated), in the *StBBX20/StBBX24* gene promoter in *Solanum tuberosum*. Moreover, in potato transgenic plants with silenced *StZPR1* expression, there are disturbances of some BBX genes’ daily oscillations, such as *StBBX5*, *StBBX9*, *StBBX18*, *StBBX20/StBBX24*, and *StBBX27* [134]. Another interesting fact is that in promoter regions of some *BBX* genes, both circadian and ABRE *cis*-elements are found among several plant species (Table 2). Due to the fact that, recently, more evidence regarding the role of BBX in stress response and circadian regulation has been accumulated, further investigation of the relation of both those pathways would contribute a lot into understanding how plants respond to stresses, specifically for further elucidation of circadian clock regulation mechanism under water-deficit conditions.

## 9. Conclusions

The circadian clock synchronizes biological events with day/night cycles. Although the clock may not be directly involved in plant-stress responses and survival, it facilitates plant fitness to privileged environmental cues and survival by synchronizing the plants’ physiological processes. ABA is the main plant hormone involved in the stomatal water-loss reaction of plants in response to humidity levels in both the rhizosphere and the boundary layer of the leaf. In the present review, we hypothesized that the circadian clock enhances fitness to water-scarcity conditions by controlling WUE by affecting stomatal movements. This improvement in fitness is due to the involvement of ABA on photoprotection and stomatal movements, and the interrelationships between circadian clocks, ABA signaling, and stomatal responses.

## Figures and Tables

**Table 1:** Drought-response findings of various plant species regarding circadian clock genes.

Species	Gene/Mutant	Drought-Response Findings	Ref.
*Populus simonii*	LHY	Decrease in expression	[94]
Soybean	*TOC1* *LUX*, *ELF4*	Down-regulation of evening-specific components of the clock	[90]
Soybean	*PRR7* *TOC1*	Expression deviated from normal	[95]
Soybean	PRR3	Drought-specific splicing pattern was observed	[95]
Rice	*OsCCA1*/*LHY*	Increased transcription at 4 a.m.	[118]
Rice	*OsPRR1*/*TOC1*	Disruption of expression	[118]
*Gossypium hirsutum*	*PRR* gene family	*GhPRR5a*, *b*, *d*, *GhPRR3a*, and *GhPRR3c* may be involved in drought response	[25]
*Arabidopsis thaliana*	DREB1/CBF		[98]
Rice	idr1-1	Increase in ROS production and ROS-scavenging enzyme	[119]

**Table 2:** List of the *BBX* genes with both ABRE and circadian “CAANNNATC” motif identified in their promoters.

Species	*BBX* Genes with ABRE and Circadian Motif	Ref.
Tomato	*BBX3*, *BBX5*, *BBX7*, *BBX17*, *BBX18*, *BBX19*, *BBX23*, *BBX26*, *BBX28*	[128]
Grapevine	*BBX3*, *BBX5*, *BBX6*, *BBX11*, *BBX12*, *BBX13*, *BBX21*	[135]
*Dendrobium officinale*	*BBX1*, *BBX4*, *BBX13*	[136]
*Phalaenopsis equestris*	*BBX03*, *BBX04*, *BBX05*, *BBX06*, *BBX07*, *BBX11*, *BBX13*, *BBX14*, *BBX16*	[136]
*Arabidopsis thaliana*	*BBX2*, *BBX14*, *BBX17*, *BBX21*	[137]
Apple	*BBX2*, *BBX3*, *BBX4*, *BBX5*, *BBX6*, *BBX8*, *BBX9*, *BBX10*, *BBX11*, *BBX19*, *BBX21*, *BBX22*, *BBX23*, *BBX25*, *BBX32*, *BBX35*, *BBX41*, *BBX43*, *BBX44*, *BBX48*	[138]
Petunia	*BBX4*, *BBX5*, *BBX6*, *BBX9*, *BBX10*, *BBX12*, *BBX22*, *BBX28*	[139]
*Arachis duranensis*	*BBX1*, *BBX11*, *BBX13*, *BBX15*, *BBX16*, *BBX22*	[140]

## Data Availability

Not applicable.
